# Development and validation of prognostic nomograms for patients with metastatic small bowel adenocarcinoma: a retrospective cohort study

**DOI:** 10.1038/s41598-022-09986-0

**Published:** 2022-04-08

**Authors:** Hanlong Zhu, Si Zhao, Tianming Zhao, Kang Jiang, Lin Miao, Mingzuo Jiang, Fangyu Wang

**Affiliations:** 1 Department of Gastroenterology and Hepatology, Affiliated Jinling Hospital, Medical School of Nanjing University, Zhongshan East Road 305, Nanjing, 210002 Jiangsu China; 2 Department of Gastroenterology, Affiliated Drum Tower Hospital, Medical School of Nanjing University, Nanjing, Jiangsu China; 3 Department of Gastroenterology, Nanjing Drum Tower Hospital, Chinese Academy of Medical Science and Peking Union Medical College, Nanjing, Jiangsu China; 4 Medical Centre for Digestive Diseases, Second Affiliated Hospital, Nanjing Medical University, Nanjing, Jiangsu China

**Keywords:** Cancer models, Gastrointestinal cancer, Risk factors, Gastrointestinal diseases, Gastrointestinal models, Gastrointestinal system

## Abstract

We aimed to explore factors associated with prognosis in patients with metastatic small bowel adenocarcinoma (SBA) as well as to develop and validate nomograms to predict overall survival (OS) and cancer-specific survival (CSS). Relevant information of patients diagnosed between 2004 and 2016 was extracted from the Surveillance, Epidemiology, and End Results (SEER) database. Nomograms for predicting 1- and 3-year OS and CSS were established with potential risk factors screened from multivariate cox regression analysis. The discrimination and accuracy of the nomograms were assessed by concordance index (C-index), calibration plots, and the area under receiver operating characteristic curve (AUC). In total, 373 SBA patients with M1 category were enrolled. Multivariate analysis revealed that age, size and grade of primary tumor, primary tumor surgery, and chemotherapy were significant variables associated with OS and CSS. The C-index values of the nomogram for OS were 0.715 and 0.687 in the training and validation cohorts, respectively. For CSS, it was 0.711 and 0.690, respectively. Through AUC, decision curve analysis (DCA) and calibration plots, the nomograms displayed satisfactory prognostic predicted ability and clinical application both in the OS and CSS. Our models could be served as a reliable tool for prognostic evaluation of patients with metastatic SBA, which are favorable in facilitating individualized survival predictions and clinical decision-making.

## Background

Small intestine malignancies are rare and fatal, accounting for approximately 3% of all gastrointestinal cancers^1–3^, of which small bowel adenocarcinoma (SBA) is the most-common histopathological type, followed by neuroendocrine tumors, lymphomas, and sarcomas^4,5^. Despite its rarity, SBA is on the rise around the world, with an annual estimated 5,300 new cases and 1,100 cancer deaths in the United States^6,7^. Advancements in capsule endoscopy, enteroscopy, cross-sectional imaging techniques, and therapeutic strategies have markedly improved detection and survival rates in recent decades^8^. However, approximately one-third of patients present with advanced disease at diagnosis, and overall survival (OS) of metastatic SBA patients remains unsatisfactory, reported 8–22 months in limited, multicenter series^9,10^. Surgical excision remains the mainstay of treatment for SBA manifesting as localized disease, while treatment for SBA patients with M1 category is at a standstill, for whom the rationales for the choice of therapeutic modalities are extrapolated from colon adenocarcinoma^11^. Therefore, it has become imperative to estimate clinicopathologic characteristics related to prognosis, thus facilitating individualized and optimal patient treatment.

To date, the American Joint Committee on Cancer (AJCC) TNM staging system has been periodically updated for prognostic evaluation of SBAs, which only takes the depth of tumor invasion (T), number of metastatic lymph nodes (N), and distant metastasis (M) into consideration. Other independent factors, however, such as age^12,13^, gender^13^, race^14^, performance status^15^, carbohydrate antigen 19-9 (CA 19-9)^16^, carcinoembryonic antigen (CEA)^15^, tumor site^17,18^, metachronous or synchronous metastasis^19^, and treatment^20,21^ can also influence the survival of SBA patients significantly. Additionally, tumor biological behavior including tumor size^22^, histological type^20^, the degree of differentiation^21^, growth pattern^18^, and metastasis pathway^18^ can affect the prognosis of SBA patients to a certain extent. Likewise, a lack of emotional support, family care, and medical insurance implicates that this category of patients with tumors would miss the optimal treatment opportunity and have a poor prognosis^23^. Consequently, the role of sociomedical support covering marital and insurance status cannot be neglected in the clinical outcome of SBA patients^24,25^. Under this circumstance, the traditional TNM staging system needs further improvement and validation, which is inadequately formulated for the prognostic prediction and may not deficiently encompass the tumor biology^26^. And a more effective and accurate model is under requirement for predicting the prognosis of patients with metastatic SBA.

Of the available and effective decision-making tools, nomogram, as an integrative graphical calculation or algorithm that incorporates biological and clinical variables, is the most widely used to predict individual prognosis in clinical investigations currently. It has been proved to be favorable and accurate compared to the TNM staging system in diverse cancers, highlighting their application as new alternatives or even new standards^27–30^, with the advantage of quantification and visualization for clinicians. To the best of our knowledge, although a prior study has reported a nomogram about cancer-specific survival (CSS) in the whole patients with SBA^13^, it did not refer to the populations with substantially poorer prognosis, nor did it conduct a subgroup analysis on this. Therefore, the present study aimed to identify demographic and tumor characteristics that influence the particular outcomes of patients with metastatic SBAs and subsequently develop nomograms to predict 1- and 3-year OS and CSS based on the Surveillance, Epidemiology, and End Result (SEER) database, which could provide more informative and representative evidence.

## Methods

### Database and patient selection

**Figure 1:**
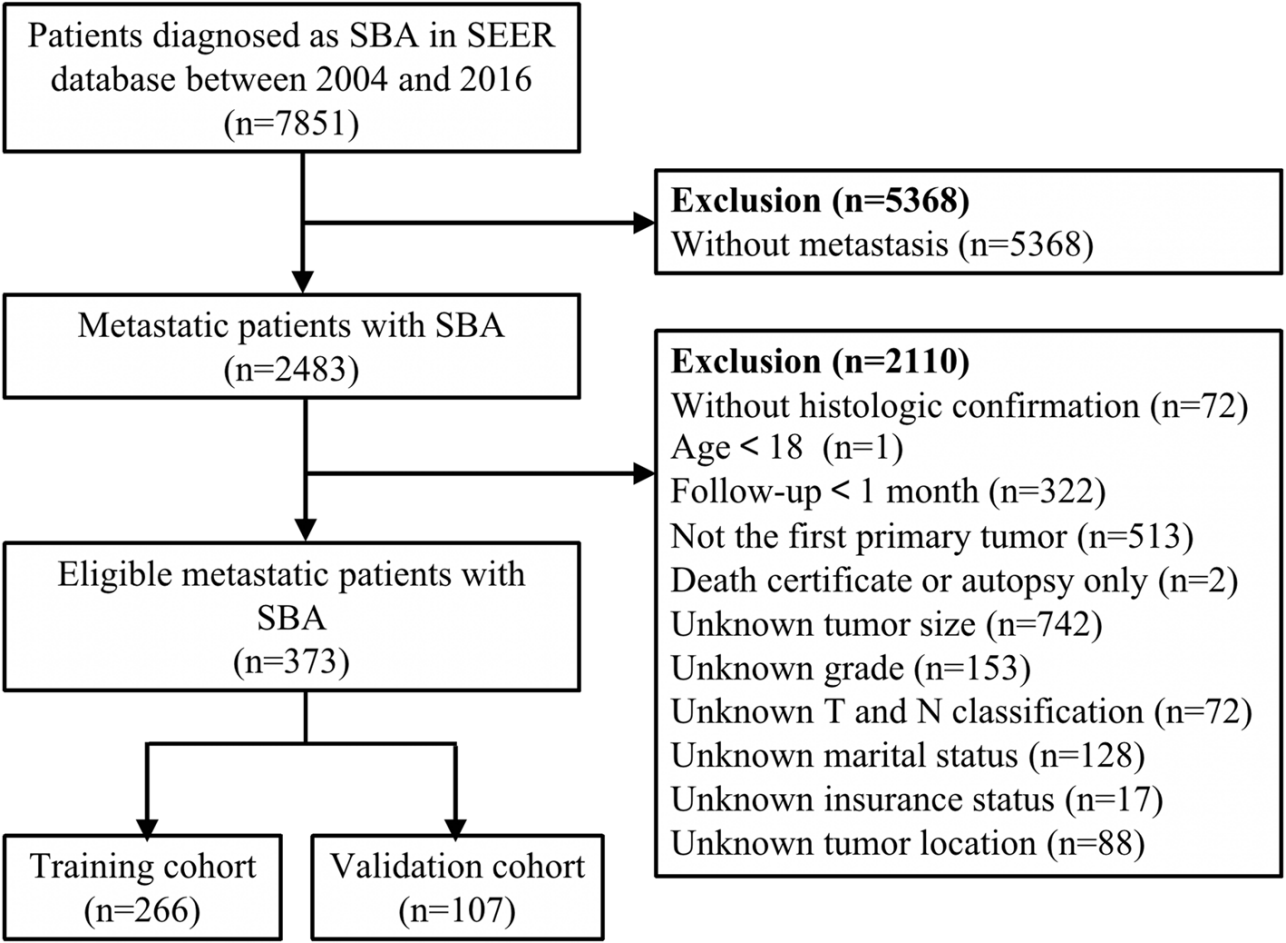
Flow diagram of eligible metastatic patients diagnosed with small bowel adenocarcinoma (SBA).

Information of patients with a histological confirmation of SBA with M1 category were obtained between 2004 and 2016 after receiving permission to access the SEER research files (accession number: 18892-Nov 2018). The SEER program is a national collaboration cancer registry, which comprehensively accumulates demographic and clinical information on associated prevalence, treatment and prognosis of various cancer types, covering up to 34% of the US population^31^. We used the following SEER variables to identify primary SBA: “Primary Site-labeled” (C17.0-duodenum, C17.1-jejunum, C17.2-ileum, or C17.9-small bowel not otherwise specified) and “Histologic Type International Classification of Diseases for Oncology, Third edition (ICD-O-3)” (histology codes: 8140, 8144, 8210, 8211, 8220, 8221, 8255, 8260, 8261, 8262, 8263, 8480, 8481, 8490, 8574, or 8576). The detailed inclusion criterias were as follows: (a) patients pathologically diagnosed with metastatic SBA from 2004 to 2016; (b) age ≥ 18 years; (c) no history of other types of malignancy; (d) survival months and follow-up information were available; (e) complete data on tumor location and size, grade, T classification, status of nodal metastasis, marital and insurance status, and treatment. Besides, patients diagnosed at the time of autopsy and death certificates were excluded. Finally, 373 SBA patients with M1 category were deemed eligible, and they were randomly divided into the training set and the validation set at a ratio of 7:3. The flowchart of patient selection was presented in Fig. 1.

### Data extraction

Individual data retrieved in our analysis included race, gender, marital and insurance status of patients, age at diagnosis, year of diagnosis, tumor location, histology, tumor size and grade, T classification of 8th AJCC staging scheme, nodal metastasis, and treatment (surgery, chemotherapy, and radiation therapy status) of primary tumor, distant metastasis site, vital status, cause-specific death classification, and survival time. For patients with no tumor resection, the T classification, nodal metastasis, and tumor size were assessed by endoscopy and imaging, such as esophagogastroduodenoscopy with endoscopic ultrasound, double balloon endoscopy, capsule endoscopy, CT, MRI, PET-CT and so on, which could evaluate the extent of local tumor invasion and lymph node involvement^32^. To maximize predictive ability, continuous age variable was further categorized into three groups based on the optimal cut-off values generated by X-tile program. In the same way, tumor size was transformed into dichotomous categorical variable. According to the best cut-off values, the age of diagnosis was grouped into three categories: ≤ 59 years old (150 cases), 60–75 years old (159 cases), and ≥ 76 years old (64 cases). Tumor size was stratified into two sets: ≤ 48 mm (218 cases,), and ≥ 49 mm (155 cases) (Fig. S1). Analogously, the categorical variables were also classified accordingly for some clinical reasons. Divorced, separated, widowed, and single patients were converted into unmarried category. Surgery was defined as two styles, including primary radical surgery (total removal of the primary site with an en bloc resection of other organs) and primary palliative surgery (excisional biopsy, laser ablation, simple or partial surgical removal of primary site, etc.) according to the SEER surgery codes for the small intestine. Treatment was classified as primary tumor surgery alone, primary tumor surgery plus chemotherapy or radiation, chemotherapy or radiation alone and none (receive no therapy). The principal outcome of interest was the probability of 1-year and 3-year OS, whereas CSS was the secondary endpoint of our study. The reason why 1-year and 3-year outcomes were chosen was that the majority of patients experienced death within 3 years. We defined OS as the duration between first diagnosis of SBA and death or the last follow-up control. CSS was measured as the interval from a positive diagnosis to death attributed to SBA (or the most recent contact data).

### Statistical analysis

A descriptive analysis of the basic characteristics of patients was conducted. Continuous variables were presented as the mean and all categorical data were reported as the number of cases with proportions. The Chi-square test and Student's t-test were used to compare the demographic and clinical parameters between the training cohort and the validation cohort. Univariate and multivariate Cox regression analysis were applied to analyze risk factors on OS and CSS along with hazard ratios (HRs) and corresponding 95% confidence intervals (CIs). Afterwards, a predictive model was constructed to predict 1-, 3-year OS and CSS on the basis of the independent prognostic variables identified from the multivariate analysis in the training cohort. It is worth mentioning that treatment did not enter into the univariate and multivariate analysis because of the collinearity between the treatment and variables such as primary tumor surgery, chemotherapy, and radiation.

Both discrimination and calibration were measured to assess the performance of the nomogram using the training set and internal validation set. The ability to discriminate between observed and predicted outcome was evaluated by Harrell's concordance index (C-index)^33^. A higher C-index indicated a superior capacity to separate patients with different survival outcomes. Similarly, the area under receiver operating characteristic (ROC) curve (AUC) was further utilized to appraise the prediction efficiency of the prognostic models. The larger the area is, the more precise the model’s predictive ability is. The calibration curves were performed according to a bootstrapped resample with 1000 iterations. A calibration plot in the 45-degree line implied a perfect model, with great concordance between the predicted and actual survival. Furthermore, the clinical value of the predictive models was reckoned with decision curve analysis (DCA) by quantifying the net benefit at distinct threshold probabilities^34^. Additionally, the total scores of each patient were calculated based on the established Cox regression model, and then patients were assigned into the low-, and high-risk groups using the X-tile program. Survival curves among two groups of patients with different prognostic risk were delineated by the Kaplan–Meier method and log-rank test.

SPSS software version 25.0 (IBM Corp, Armonk, NY), X-tile software version 3.6.1 (Rimm Laboratory, Yale School of Medicine, New Haven, CT, USA), and R software version 3.3.0 (Institute for Statistics and Mathematics, Vienna, Austria) were used for above statistical analysis, and statistical significance would be observed when P value was below 0.05 in a two-tailed test.

### Ethics statement

The current study was based on available SEER database in which data contained unidentifiable patient information and were publicly retrieved. Therefore, the study approval was exempted by the institutional review board review. This article does not contain any studies with human participants performed by any of the authors. All methods were performed in accordance with relevant guidelines and regulations.

### Ethics approval and consent to participate

Institutional review board approval was not needed for this study as it utilized publically available data.

## Results

### Clinicopathologic characteristics of patients

The demographic and clinicopathological features of the total cohort are presented in Table 1, and there was no statistically significant difference between the training and validation sets. Among the eligible patients, the mean age was 62 years (20–91 years). The majority of patients were white (65.1%), married (61.9%) and insured (85.0%) individuals, with a greater percentage of smaller tumor size (≤ 48 mm). The distribution of different tumor locations was not even. 58.2% of patients were located in the distal site (jejunum and ileum), with lower prevalence of duodenum (41.8%). Overall, the most frequent organ of metastasis was the liver (27.1%). For tumor histology, conventional adenocarcinoma (87.9%) was the most common, followed by mucinous adenocarcinoma (6.4%) and signet ring cell carcinoma (5.7%). Besides, the cohorts were also in unequally distribution of T classification: T1/T2 (9.9%), and T3/T4 (90.1%). In both sets, they were far more likely to happen nodal metastasis. Generally, 268 patients performed primary tumor surgery while 105 have not in the whole cohort, of whom 55 underwent primary radical surgery and 213 had primary palliative surgery. As to adjuvant treatment, there were nearly two thirds people receiving chemotherapy (263/373, 70.5%).

**Table 1:** Patient characteristics in the study.

Characteristic (N, %)	Total n = 373	Validation cohort n = 107	Training cohort n = 266	*p* value
Age (years), mean	62.25 ± 13.69	62.57 ± 14.00	62.12 ± 13.58	0.775
**Age group, years**				0.712
≤ 59	150 (40.2)	41 (38.3)	109 (41.0)	
60–75	159 (42.6)	45 (42.1)	114 (42.9)	
≥ 76	64 (17.2)	21 (19.6)	43 (16.1)	
**Race**				0.090
White	264 (65.1)	69 (64.5)	195 (73.3)	
Non-white	109 (34.9)	38 (35.5)	71 (26.7)	
Gender				0.167
Male	216 (57.9)	56 (52.3)	160 (60.2)	
Female	157 (42.1)	51 (47.7)	106 (39.8)	
**Marital status**				0.593
Married	231 (61.9)	64 (59.8)	167 (62.8)	
Unmarried	142 (38.1)	43 (40.2)	99 (37.2)	
**Insurance status**				0.792
Insured	317 (85.0)	91 (85.0)	226 (85.0)	
Any Medicaid	42 (11.3)	13 (12.2)	29 (10.9)	
Uninsured	14 (3.7)	3 (2.8)	11 (4.1)	
**Diagnosis time**				0.582
2004–2010	130 (34.9)	35 (32.7)	95 (35.7)	
2011–2016	243 (65.1)	72 (67.3)	171 (64.3)	
**Histology**				0.355
Mucinous adenocarcinoma	24 (6.4)	9 (8.4)	15 (5.6)	
Signet ring cell carcinoma	21 (5.7)	8 (7.5)	13 (4.9)	
Conventional adenocarcinoma	328 (87.9)	90 (84.1)	238 (89.5)	
**Primary tumor site**				0.384
Duodenum	156 (41.8)	41 (38.3)	115 (43.2)	
Jejunum/Ileum	217 (58.2)	66 (61.7)	151 (56.8)	
**Primary tumor size**				0.292
≤ 48 mm	218 (58.4)	58 (54.2)	160 (60.2)	
≥ 49 mm	155 (41.6)	49 (45.8)	106 (39.8)	
**Metastatic site**				0.501
Liver	101 (27.1)	25 (23.4)	76 (28.6)	
Lung	13 (3.5)	3 (2.8)	10 (3.8)	
Others*	259 (69.4)	79 (73.8)	180 (67.6)	
**T classification**				0.814
T1/T2	37 (9.9)	10 (9.3)	27 (10.2)	
T3/T4	336 (90.1)	97 (90.7)	239 (89.8)	
**Nodal metastasis**				0.944
Negative	121 (32.4)	35 (32.7)	86 (32.3)	
Positive	252 (67.6)	72 (67.3)	180 (67.7)	
**Grade**				0.405
G1/G2	180 (48.3)	48 (44.9)	132 (49.6)	
G3/G4	193 (51.7)	59 (55.1)	134 (50.4)	
**Primary tumor surgery**				0.960
No	105 (28.2)	29 (27.1)	76 (28.5)	
Radical surgery	55 (14.7)	16 (15.0)	39 (14.7)	
Palliative surgery	213 (57.1)	62 (57.9)	151 (56.8)	
**Chemotherapy**				0.163
No	110 (29.5)	26 (24.3)	84 (31.6)	
Yes	263 (70.5)	81 (75.7)	182 (68.4)	
**Radiation**				0.742
No	346 (92.8)	100 (93.5)	246 (92.5)	
Yes	27 (7.2)	7 (6.5)	20 (7.5)	
**Treatment**				0.574
Primary tumor surgery alone	78 (20.9)	19 (17.8)	59 (22.2)	
Primary tumor surgery + Chemotherapy/Radiation	190 (50.9)	59 (55.1)	131 (49.3)	
Chemotherapy/Radiation alone	81 (21.8)	24 (22.4)	57 (21.4)	
None	24 (6.4)	5 (4.7)	19 (7.1)	

*Others: bone, brain, distant lymph node, and other sites.

### Development and construction of the nomogram

**Figure 2:**
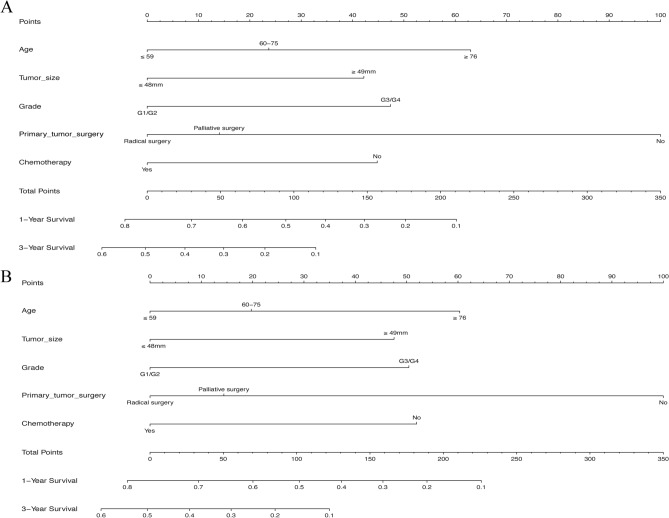
Nomograms to predict the probability of 1- and 3-year OS (**A**) and CSS (**B**). OS, overall survival; CSS, cancer-specific survival.

As shown in Tables 2 and 3, age at diagnosis (*P* = 0.000, both), race (*P* = 0.013, *P* = 0.018, respectively), primary tumor site (*P* = 0.004, *P* = 0.016, respectively), tumor size (*P* = 0.001, *P* = 0.000, respectively), metastatic site (*P* = 0.003, *P* = 0.004, respectively), T classification (*P* = 0.007, *P* = 0.019, respectively), grade (*P* = 0.001, *P* = 0.002, respectively), primary tumor surgery (*P* = 0.000, both), chemotherapy (*P* = 0.004, *P* = 0.003, respectively), and radiation (*P* = 0.016, *P* = 0.010, respectively) were significantly connected with OS and CSS by univariate analysis for the training cohort. Meanwhile, taking the results of multivariate analysis into account, the following five independent predictive variables were integrated into the prognostic nomogram of 1- and 3-year OS (Fig. 2A) and CSS (Fig. 2B), including age at diagnosis (*P* = 0.017, *P* = 0.042, respectively), tumor size (*P* = 0.005, *P* = 0.004, respectively), grade (*P* = 0.002, *P* = 0.003, respectively), primary tumor surgery (*P* = 0.002, *P* = 0.005, respectively), and chemotherapy (*P* = 0.004, *P* = 0.002, respectively). Each risk factor was assigned a score on a points scale, and by projecting the total scores to the bottom scale, the probability of 1-, and 3-year OS and CSS can be easily predicted.

**Table 2:** Univariate and multivariate analyses of overall survival for the training cohort.

Characteristic (n = 266)	Univariate analysis		Multivariate analysis	
	HR (95% CI)	*p* value	HR (95% CI)	*p* value
**Age group, years**		0.000		0.017
≤ 59	Reference		Reference	
60–75	1.393 (1.029–1.886)	0.032	1.292 (0.943–1.769)	0.110
≥ 76	2.474 (1.684–3.634)	0.000	1.815 (1.199–2.748)	0.005
**Race**		0.013		0.088
White	Reference		Reference	
Non-white	0.666 (0.483–0.918)	0.013	0.747 (0.535–1.045)	0.088
**Gender**		0.109		
Male	Reference			
Female	0.795 (0.601–1.052)	0.109		
**Marital status**		0.727		
Married	Reference			
Unmarried	0.951 (0.717–1.261)	0.727		
**Insurance status**		0.501		
Insured	Reference			
Any Medicaid	0.749 (0.461–1.217)	0.244		
Uninsured	0.926 (0.472–1.814)	0.822		
**Diagnosis time**		0.585		
2004–2010	Reference			
2011–2016	0.926 (0.702–1.221)	0.585		
**Histology**		0.129		
Mucinous adenocarcinoma	Reference			
Signet ring cell carcinoma	2.320 (0.973–5.531)	0.058		
Conventional adenocarcinoma	1.900 (0.971–3.716)	0.061		
Primary tumor site		0.004		0.709
Duodenum	Reference		Reference	
Jejunum/Ileum	0.669 (0.508–0.881)	0.004	1.083 (0.711–1.650)	0.709
**Primary tumor size**		0.001		0.005
≤ 48 mm	Reference		Reference	
≥ 49 mm	1.622 (1.229–2.140)	0.001	1.519 (1.135–2.035)	0.005
**Metastatic site**		0.003		0.082
Liver	Reference		Reference	
Lung	1.648 (0.841–3.232)	0.146	1.367 (0.654–2.859)	0.406
Others	0.678 (0.498–0.922)	0.013	0.753 (0.545–1.041)	0.086
**T classification**		0.007		0.190
T1/T2	Reference		Reference	
T3/T4	0.554 (0.361–0.849)	0.007	0.730 (0.456–1.168)	0.190
**Nodal metastasis**		0.758		
Negative	Reference			
Positive	0.956 (0.717–1.275)	0.758		
**Grade**		0.001		0.002
G1/G2	Reference		Reference	
G3/G4	1.564 (1.190–2.056)	0.001	1.560 (1.172–2.077)	0.002
**Primary tumor surgery**		0.000		0.002
No	Reference		Reference	
Radical surgery	0.384 (0.243–0.606)	0.000	0.409 (0.238–0.705)	0.001
Palliative surgery	0.450 (0.329–0.615)	0.000	0.456 (0.281–0.741)	0.002
**Chemotherapy**		0.004		0.004
No	Reference		Reference	
Yes	0.654 (0.488–0.875)	0.004	0.630 (0.458–0.865)	0.004
**Radiation**		0.016		0.848
No	Reference		Reference	
Yes	1.825 (1.118–2.978)	0.016	0.946 (0.538–1.663)	0.848

Others*: bone, brain, distant lymph node, and other sites; HR, hazard ratio; CI, confidence interval.

**Table 3:** Univariate and multivariate analyses of cancer-specific survival for the training cohort.

Characteristic (n = 266)	Univariate analysis		Multivariate analysis	
	HR (95% CI)	*p* value	HR (95% CI)	*p* value
**Age group, years**		0.000		0.042
≤ 59	Reference		Reference	
60–75	1.318 (0.969–1.791)	0.078	1.226 (0.892–1.686)	0.209
≥ 76	2.353 (1.591–3.481)	0.000	1.715 (1.125–2.615)	0.012
**Race**		0.018		0.124
White	Reference		Reference	
Non-white	0.676 (0.488–0.935)	0.018	0.766 (0.546–1.076)	0.124
**Gender**		0.174		
Male	Reference			
Female	0.821 (0.619–1.091)	0.174		
**Marital status**		0.577		
Married	Reference			
Unmarried	0.921 (0.690–1.229)	0.577		
**Insurance status**		0.597		
Insured	Reference			
Any Medicaid	0.778 (0.479–1.264)	0.311		
Uninsured	0.959 (0.489–1.880)	0.903		
**Diagnosis time**		0.419		
2004–2010	Reference			
2011–2016	0.891 (0.672–1.180)	0.419		
**Histology**		0.181		
Mucinous adenocarcinoma	Reference			
Signet ring cell carcinoma	2.139 (0.882–5.187)	0.092		
Conventional adenocarcinoma	1.819 (0.929–3.561)	0.081		
**Primary tumor site**		0.016		0.580
Duodenum	Reference		Reference	
Jejunum/Ileum	0.709 (0.535–0.939)	0.016	1.128 (0.737–1.725)	0.580
**Primary tumor size**		0.000		0.004
≤ 48 mm	Reference		Reference	
≥ 49 mm	1.659 (1.252–2.199)	0.000	1.549 (1.151–2.084)	0.004
**Metastatic site**		0.004		0.073
Liver	Reference		Reference	
Lung	1.734 (0.882–3.408)	0.111	1.395 (0.665–2.924)	0.379
Others	0.689 (0.503–0.943)	0.020	0.747 (0.536–1.040)	0.084
**T classification**		0.019		0.268
T1/T2	Reference		Reference	
T3/T4	0.586 (0.376–0.915)	0.019	0.759 (0.466–1.237)	0.268
**Nodal metastasis**		0.961		
Negative	Reference			
Positive	0.993 (0.739–1.333)	0.961		
Grade		0.002		0.003
G1/G2	Reference		Reference	
G3/G4	1.559 (1.181–2.059)	0.002	1.559 (1.165–2.087)	0.003
**Primary tumor surgery**		0.000		0.005
No	Reference		Reference	
Radical surgery	0.414 (0.260–0.658)	0.000	0.432 (0.248–0.752)	0.003
Palliative surgery	0.479 (0.347–0.660)	0.000	0.474 (0.288–0.779)	0.003
**Chemotherapy**		0.003		0.002
No	Reference		Reference	
Yes	0.636 (0.473–0.855)	0.003	0.607 (0.440–0.837)	0.002
**Radiation**		0.010		0.891
No	Reference		Reference	
Yes	1.911 (1.170–3.122)	0.010	1.040 (0.590–1.834)	0.891

Others*: bone, brain, distant lymph node, and other sites; HR, hazard ratio; CI, confidence interval.

### Validation of the nomogram

**Figure 3:**
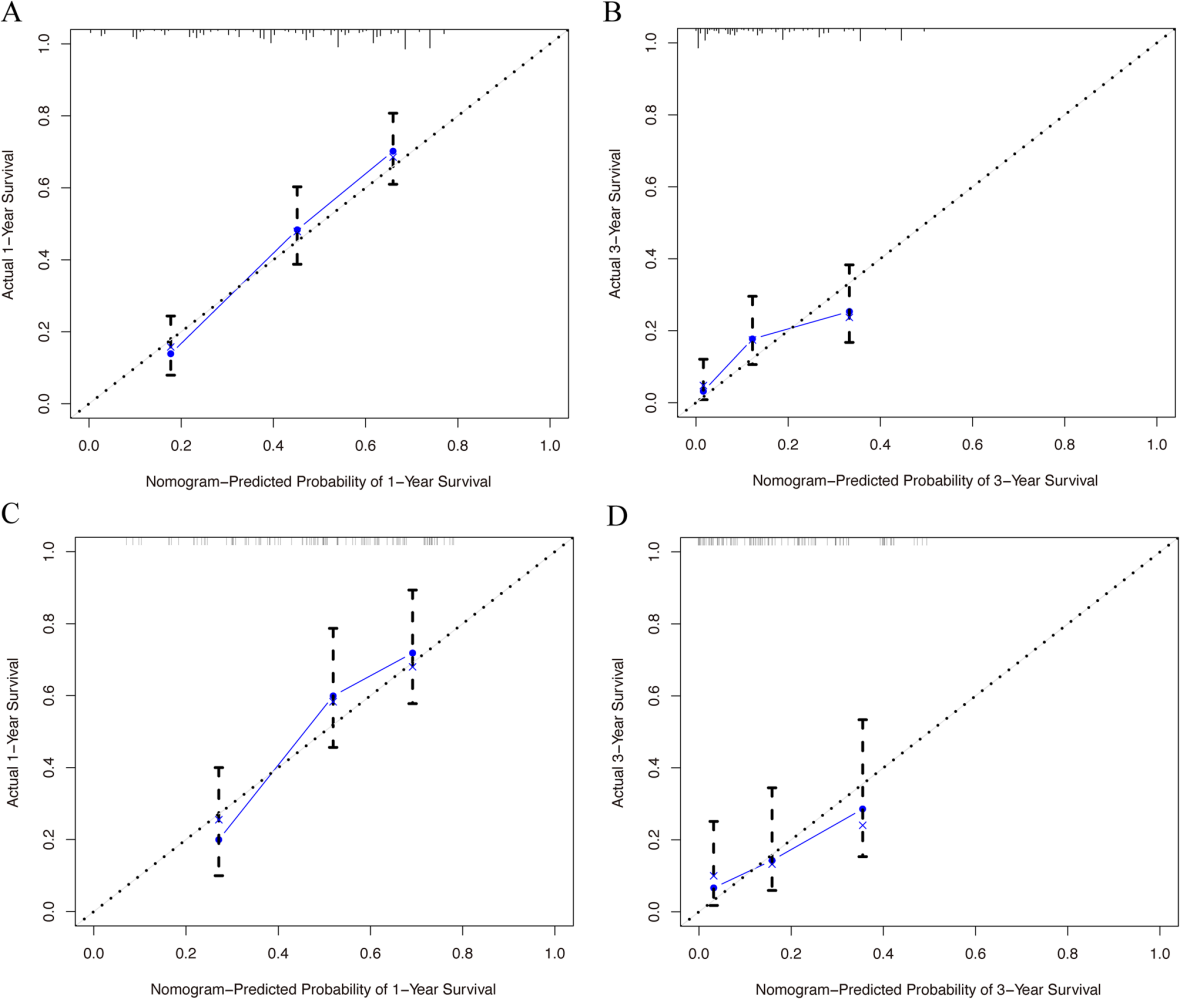
Calibration plot of the nomogram for 1- and 3-year OS: (**A**) at 1 year in the training set; (**B**) at 3 year in the training set; (**C**) at 1 year in the validation set; (**D**) at 3 year in the validation set. OS, overall survival.

**Figure 4:**
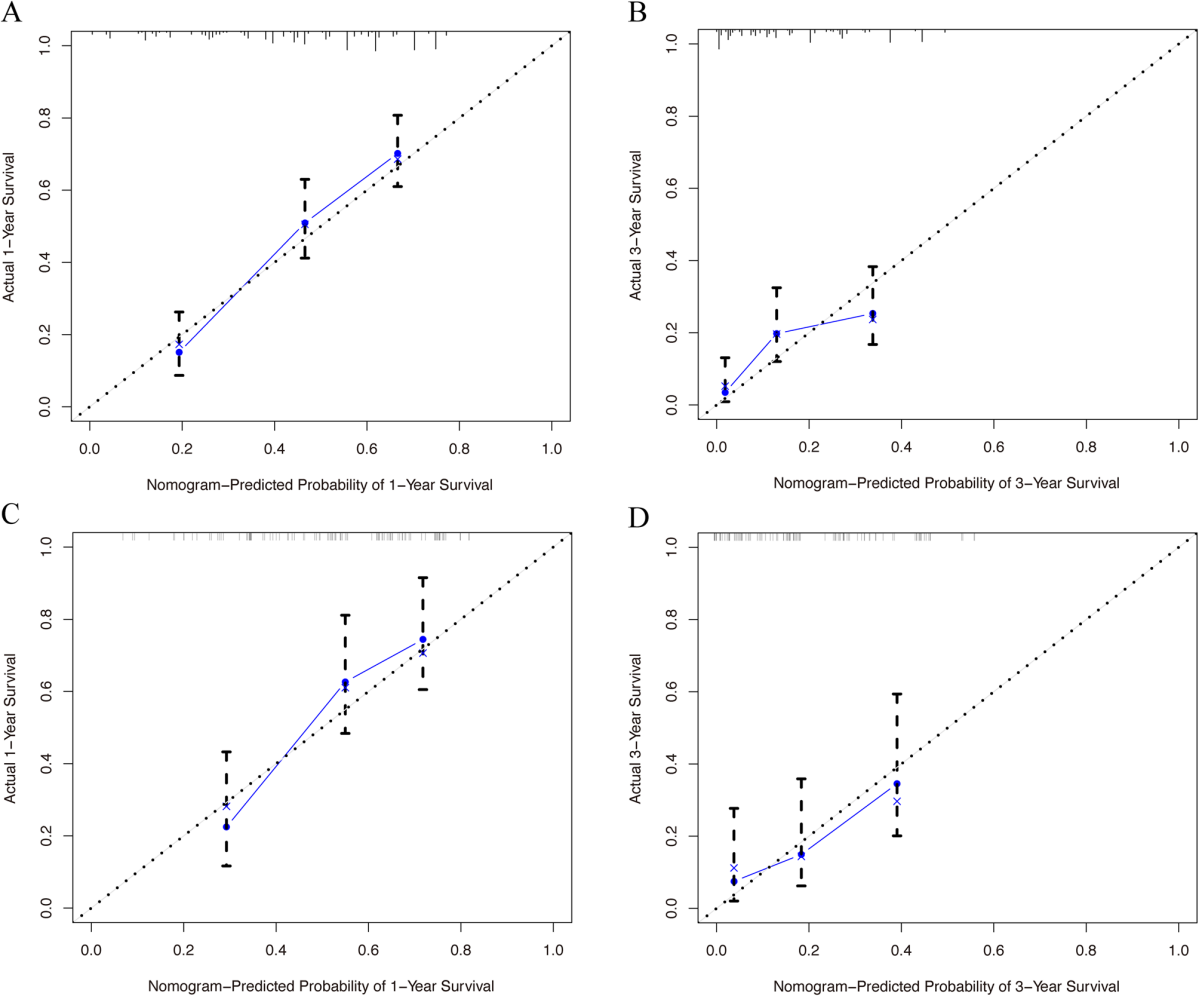
Calibration plot of the nomogram for 1- and 3-year CSS: (**A**) at 1 year in the training set; (**B**) at 3 year in the training set; (**C**) at 1 year in the validation set; (**D**) at 3 year in the validation set. CSS, cancer-specific survival.

**Figure 5:**
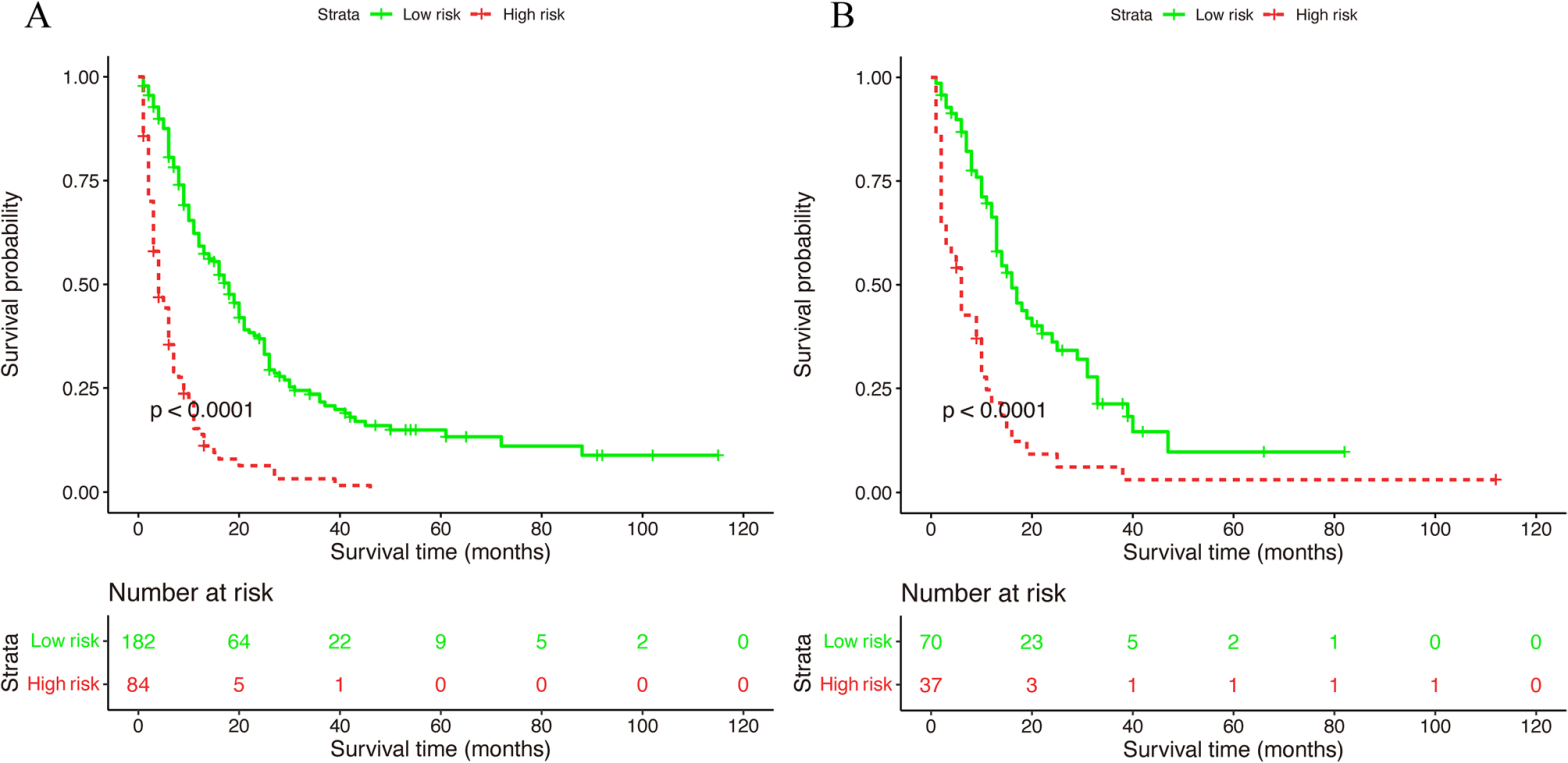
Kaplan–Meier survival curves of nomogram for OS in the training cohort (**A**) and validation cohort (**B**). OS, overall survival.

**Figure 6:**
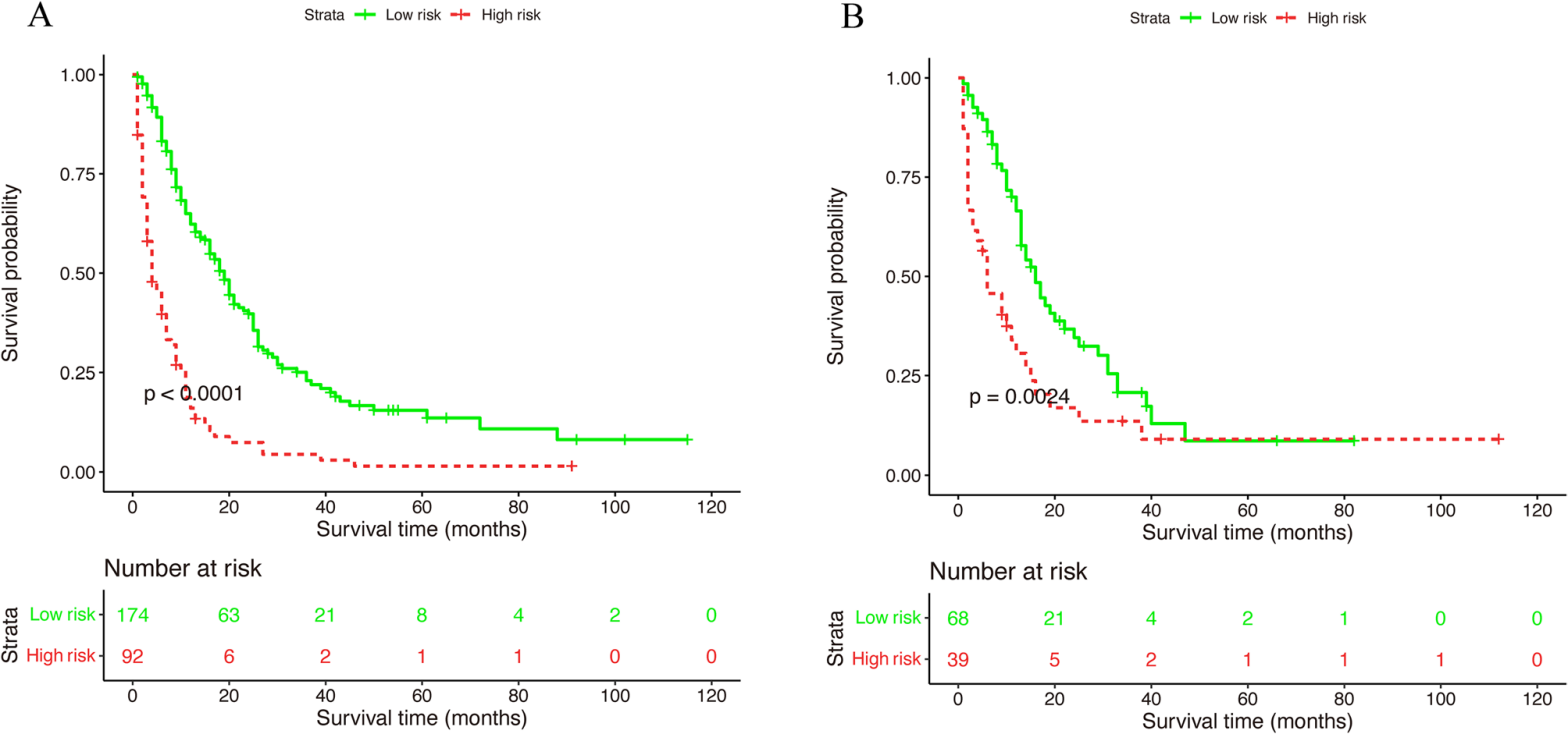
Kaplan–Meier survival curves of nomogram for CSS in the training cohort (**A**) and validation cohort (**B**). CSS, cancer-specific survival.

The training set manifested that the C-index values to appraise OS and CSS were 0.715 (95% CI, 0.711–0.719) and 0.711 (95% CI, 0.707–0.715), respectively. Analogously, for the internal validation cohort, the predictive value of OS was 0.687 (95% CI, 0.680–0.694), and the C-index for prediction of CSS was 0.690 (95% CI, 0.683–0.697). The 1- and 3-year AUC values for OS were 0.800 and 0.689, respectively, in the training cohort, and 0.700 and 0.640 in the validation cohort (Fig. S2). Similarly, the 1- and 3-year AUC values for CSS were 0.800, 0.685, 0.700, and 0.638 in the training and validation sets, respectively (Fig. S3). These results exhibited favorable survival predictive ability of nomograms. Then we conducted the calibration of the nomograms with a bootstrap sampling for 1000 times, and the calibration plots in both training and validation cohorts displayed an excellent correlation between the predicted and observed survival probability (Figs. 3 and 4). Specially, the DCA also presented that the developed models in predicting OS and CSS showed a larger net benefit with a wider range of threshold probabilities in the analysis (Figs. S4 and S5). In summary, the nomograms for metastatic SBA showed considerable discriminative and calibrating abilities. Moreover, we divided all 373 patients into low-risk group, and high-risk group according to individual scores, and plotted the Kaplan–Meier curves. As shown in Fig. 5, the median OS of patients among two groups were 18, and 4 months (*P* < 0.0001), respectively, in the training set, and 16, and 6 months (*P* < 0.0001), respectively, in the validation set. While for CSS (Fig. 6), compared with lower risk group, patients who presented with higher risk had worse survival outcomes in both cohorts (19 and 4 months in the training set and 16 and 6 months in the validation set; *P* < 0.0001 and *P* = 0.0024, respectively), illustrating that there was no apparent difference in utilization of the models between the training and validation groups.

## Discussion

In view of the epidemiological facts, the annual incidence of SBA is steadily on the rise although it is a relatively rare tumor. However, it was noteworthy that far fewer studies on its prognosis was reported for patients with distant metastases of SBA in comparison to their counterparts without metastasis, while distant metastasis is extremely essential for treatment selection and survival assessment. Traditional TNM staging system is commonly used for the prognostication of metastatic SBA, but it solely considers the anatomical scope of the disease without taking biofunctional heterogeneity into account, leading to an imprecise evaluation of prognosis, particularly in patients with incurable tumors^35^. Recent years, clinicians have been continually wrestling with obstacles regarding the way to optimally incorporate established and novel prognostic variables alongside anatomic stage into personalized estimation of clinical events. Accordingly, the nomogram, a graphical presentation of a mathematical model, is developed by combining available baseline clinical and laboratory information for the identification of the possibility of outcomes. It has been reported that nomograms achieve more superior predictive precision and prognostic value than the existing tumor staging system for numerous cancers^30,36,37^. Hence, it is of great significance to conduct an efficient nomogram model, which will facilitate survival predictions of SBA patients with M1 category and enable the administration of individualized therapies.

In this study, a novel nomogram model was established by incorporating these putative prognostic factors to predict the 1-, and 3-year OS and CSS rates of SBA patients with M1 category. The final parameters incorporated in the predictive model were age, size and grade of primary tumor, primary tumor surgery, and chemotherapy, which are easily available and measurable during diagnosis and treatment. Additionally, the nomogram indicated excellent discrimination and showed superior clinical usability throughout the survival as assessed by DCA.

It is widely known that age is an important variable related with different prognosis in malignancies^30,38^. As demonstrated in the nomogram, patients older than 76 years would have an increasing risk of death in comparison to younger patients, and this result is consistent with the previous study^11^. The potential mechanism of the correlation we found might be that some factors associated with age, including lower immune response and higher levels of chronic inflammation, may affect the survival of metastatic patients^39,40^. Moreover, the current study found that patients with a duodenal primary tumor location suffered worse survival than patients with distal adenocarcinoma in univariate analysis, but not in multivariate analysis. Meanwhile, tumor size and tumor grade were identified as predictors for the OS and CSS of metastatic SBA patients, with survival being worse in patients with lager tumor size or higher tumor grade. It has been reported that this two factors play crucial roles in the prognosis of SBA by several studies^3,25^. Surprisingly, univariate analysis indicated that a lower T category was associated with a worse prognosis in our study. Generally, tumors with higher T categories mean deeper infiltration depth and they are more likely to experience a poorer outcome. As reported in the literature, the T category served as an independent prognostic factor for SBA, and advanced T category was correlated with significantly inferior survival^12,41^. The differences exist in sample size and characteristics of study population between studies might be underlying explanations for the disparate findings. For example, compared to T3/T4 category, there were more elderly patients and fewer patients who subjected primary tumor surgeries in T1/T2 category (data not shown), which could lead to the risk of confounding and selection bias. To eliminate any potential confounders, the current study also excluded the confounding effects of these factors by using multivariate analysis. Notably, research in the form of randomized controlled trials with balanced characteristics is desperately required to fill this knowledge gap.

Accumulating evidence revealed that sociomedical support, including marital and insurance status, can impact the mental health and prognosis of patients with tumors^23,42^. Several retrospective studies have demonstrated that uninsured cancer patients were correlated with a higher risk of all-cause mortality and cancer-specific mortality in Eastern countries^43,44^. And a SEER-based study conducted by Wang et al. has also shown that patients with SBA with insurance coverage had a more favorable survival compared with medicaid and uninsured patients in the United States^24^. Analogously, another recent study of 6747 SBA patients has observed that married patients enjoyed a significantly better OS and CSS compared with unmarried patients^25^. In contrast, we were not able to detect any such association in the present study. This seemingly paradoxical phenomenon might be related to the different study populations. This study was limited to a cohort of SBA patients with M1 category, which is the most aggressive and malignant subtype of SBA with the worst outcome. One possible explanation is that auxiliary sociomedical support has little effect on the prognostic outcome of advanced disease. Thus, the relationship between marital and insurance status and metastatic SBA survival remains to be further explored.

Some clinical research has indicated that the liver is the commonest organ for metastatic spread from SBA^45,46^, which corresponded to our observations. Furthermore, in line with a prior report^10^, we confirmed that the metastatic site was not an independent prognostic factor for OS and CSS based on multivariable analysis. Due to the inherent defects of SEER database, we could not evaluate the prognostic value of the size of the metastasized tumor in the setting of metastatic SBA. Consequently, further studies with more representative samples are warranted to add this knowledge in the future.

It is noteworthy that lymph node status is an important prognostic indicator of SBA^47^. However, the reliability of positive lymph nodes staging scheme has been questioned in recent years owing to the absent consideration of the numbers of negative and total lymph nodes retrieved^48^. Accumulating evidence has declared that adequate lymph nodes histopathological assessment would translate into more dependable pathologic staging^22,49^. Subsequently, lymph node ratio (LNR) and log odds of positive lymph nodes (LODDS) were proposed, which have shown a better performance than the numbers of positive lymph nodes regarding the prognosis of patients with SBA^50^. Zhou et al. compared the impact of positive lymph nodes, LNR, and LODDS on SBA survival from the SEER database and international multicentre hospitals^48^. They concluded that LODDS scheme showed its prognostic superiority over the LNR or positive lymph nodes schemes for SBA patients, suggesting the auxiliary of the LODDS scheme to lymph node staging systems in the future revisions of AJCC manual^48^. In the current study, we only evaluated the status of nodal metastasis without the specified N classification because there are too many missing values in the SEER database. The reason for missing values is complex and multifactorial. The first one stems from the fact that the SEER database only started recording information on the number of metastatic lymph nodes in 2010. In addition, this may be related to inadequate lymph node sampling in the earlier studies of SBA. Hence, our predictive models should be further consummated by prospective multicenter studies with detailed lymph node information.

Besides the factors mentioned above, we need to highlight the significant contribution of treatment, which plays an essential role in improving survival outcomes. With regard to the treatment patterns, patients receiving primary tumor surgery combined with chemotherapy or radiation made up a larger proportion in advanced SBA, followed by chemotherapy or radiation alone, and primary tumor surgery alone in the current research. However, Puccini et al. once pointed out that the management of advanced SBA (unresectable or metastatic) is mainly based on systemic treatment, although no randomized studies have been conducted to prove the benefit of systemic chemotherapy in patients with metastatic disease^51^. Of note, the predicted benefit of surgery on OS and CSS was noticed in SBA patients with M1 category. This is in accordance with the former study^21^, in which surgical resection of metastatic SBA conferred survival benefit in the whole cohort and majority of the subgroups. Nakanoko et al. reported that surgery, as a palliative treatment, might provide favorable prognosis even in the metastatic or recurrent setting^52^. Nevertheless, a previous study reported that primary tumor resection was generally not recommended in this metastatic setting except in cases of uncontrolled bleeding, perforation, or acute bowel obstruction^51^. As a result, it might be indispensable to validate the potential advantage of resection of primary tumor using a prospective study with a large population.

Although benefit of chemotherapy relied on lower level of evidence in our study, postoperative and definitive chemotherapy is still controversial. There is currently no standardized first-line chemotherapy scheme in advanced SBA, as a result of lacking randomized controlled trials comparing the different chemotherapy protocols^9^. In terms of survival advantage, Aydin et al. recommended chemotherapy in metastatic or locally advanced unresectable SBA^9^. This conclusion also requires multi-centered and prospective studies involving adequate sample sizes to suggest a therapy modality for advanced SBAs. French intergroup clinical practice guidelines suggested that fluoropyrimidine combination, such as 5-Fluorouracil or capecitabine plus oxaliplatin or cisplatin should be considered^53^. More recently, a published trial involved by a multi-institutional data registry tested the role of the combination of cytoreductive surgery (CRS) with hyperthermic intraperitoneal chemotherapy (HIPEC) in SBA patients with peritoneal metastases^54^. In this study, 152 patients obtained the 5-year survival rate of 30.8% associated to a median OS of 32 months and showed acceptable safety, proving that CRS plus HIPEC could be regarded as a new treatment option for some selected patients with peritoneal metastases from SBA. Still, the detailed information of specific chemotherapy regimen is not captured by this database. This covariate might impact on the prognosis of patients with metastatic SBA and our results could not be adjusted for these variables. More prospective well-defined cohorts are required to refine this.

There are several potential limitations that should be noted when expounding the results of our study. First, considering the retrospective nature of the SEER database, selection bias might be virtually brought in, which may lead to our observations. The second limitation stemmed from the lack of several clinicopathological parameters and treatment variables concerning comorbidities, performance status, LNR, LODDS, and biological data that have been reported as predictive factors for metastatic SBA patients. Moreover, some haematological indexes, such as elevated serum levels of lactate dehydrogenase, CA 19-9 and CEA as well as synchronous metastases were proven to be associated with poor prognosis^16,19^. Unfortunately, the SEER database did not have specific information about plasma assay and metachronous or synchronous metastasis. These variables could be an essential complement to the existing stage systems and this will be a major part of our future research. Therefore, we felt sorry that we were incapable to effectively evaluate these variables. Furthermore, we were also unable to conduct independent external validation which might strengthen the mathematical basis of predictions. In a word, the findings of the presented study could be more persuasive and instructive if the predictive model was performed by multicenter external validation with a greater amount of clinical sampling, which would efficaciously prove whether our results are more-widely acceptable and applicable in clinical practice.

## Conclusions

In summary, for patients with metastatic SBAs, we constructed nomograms to predict the OS and CSS for the first time. The validation process manifested that our models showed good discrimination and calibration, suggesting that it could be beneficial for clinicians to identify personalized treatment and survival in the metastatic population. However, the nomograms can be further optimized by exploiting potentially important factors unavailable in the database and performing external validation by independent, high-quality, and large-quantity cohort.

## Supplementary Information

Supplementary Information.

## Data Availability

The data were abstracted from the Surveillance, Epidemiology, and End Results (SEER) database. This is an open database. (https://seer.cancer.gov).

## Abbreviations

SBA: Small bowel adenocarcinoma
OS: Overall survival
CSS: Cancer-specific survival
SEER: Surveillance, Epidemiology, and End Results
C-index: Concordance index
ROC: Receiver operating characteristic
AUC: Area under the curve
DCA: Decision curve analysis
AJCC: American Joint Committee on Cancer
T: The depth of tumor invasion
N: Number of metastatic lymph nodes
M: Distant metastasis
HRs: Hazard ratios
CIs: Confidence intervals
LNR: Lymph node ratio
LODDS: Log odds of positive lymph nodes
CA 19-9: Carbohydrate antigen 19-9
CEA: Carcinoembryonic antigen
CRS: Cytoreductive surgery
HIPEC: Hyperthermic intraperitoneal chemotherapy
