# Supplementary material for: Development and validation of prognostic nomograms for patients with metastatic small bowel adenocarcinoma: a retrospective cohort study
Source: Sci Rep. 2022 Apr 8;12:5983. doi: 10.1038/s41598-022-09986-0 (PMC8993898; doi:10.1038/s41598-022-09986-0)
Supplement: Supplementary file 1 — Supplementary Information. [file 41598_2022_9986_MOESM1_ESM.docx]

Supplementary information for

**Development and validation of prognostic nomograms for patients**

**with metastatic small bowel adenocarcinoma: A retrospective cohort study**


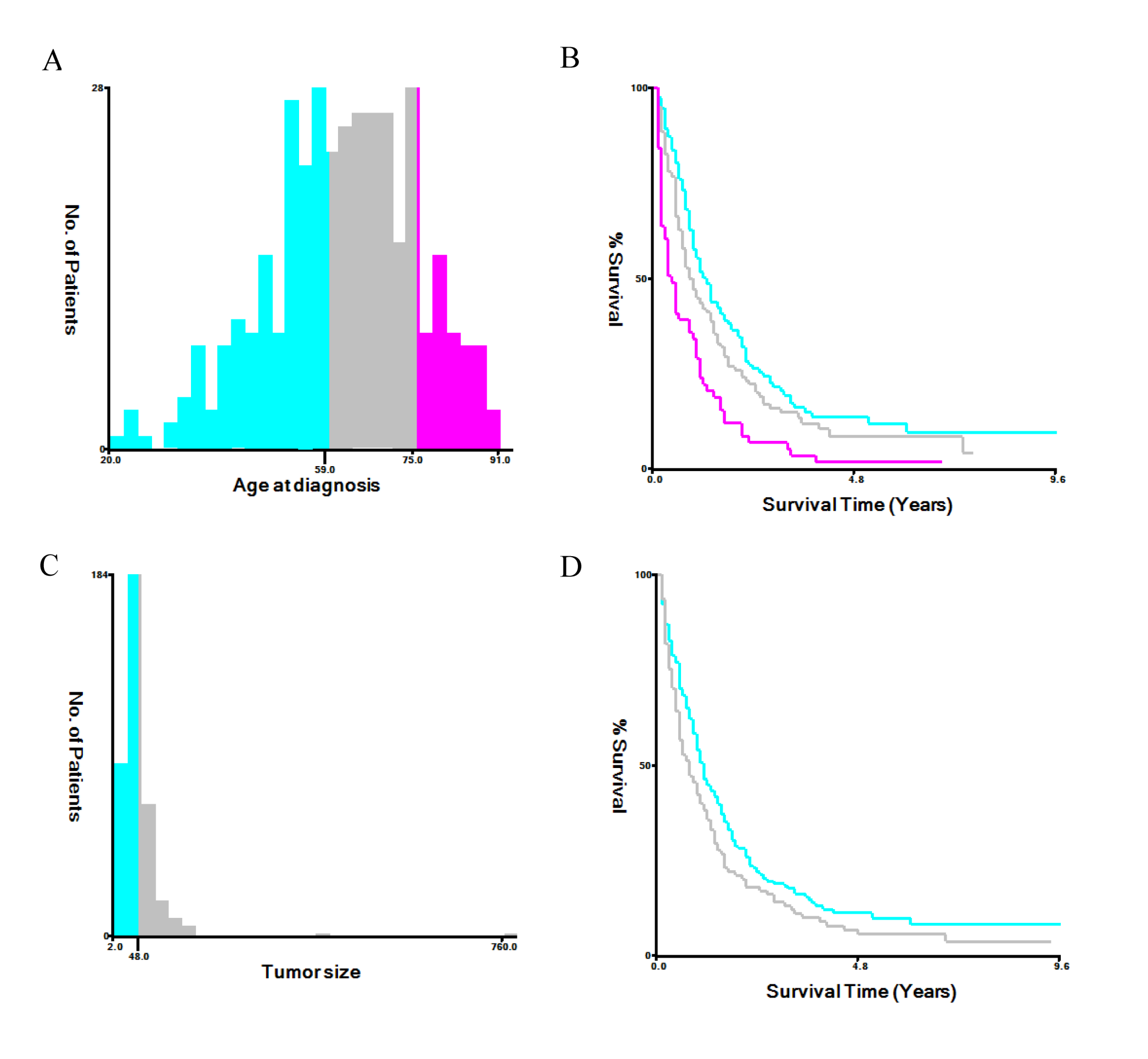


**Figure S1** Selection of the best cut-off point for Age and tumor size. (A, C) Histogram showing the best cut-off point; (B, D) Keplan-Meier curve corresponding to the cut-off point.


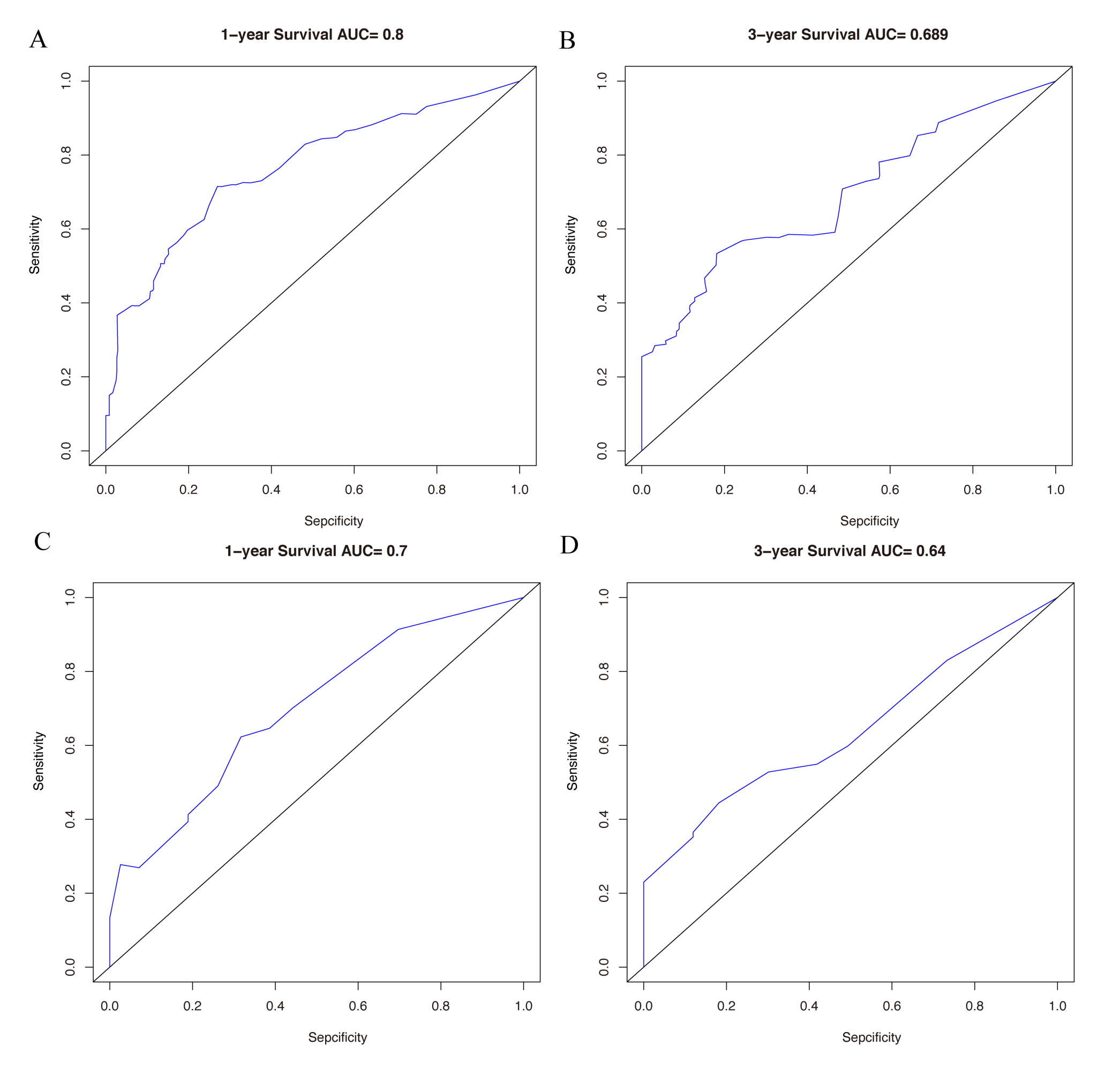


**Figure S2** The AUC values of ROC predicting for OS: (A) 1-year survival rates of the nomogram in the training set; (B) 3-year survival rates of the nomogram in the training set; (C) 1-year survival rates of the nomogram in the validation set; (D) 3-year survival rates of the nomogram in the validation set. OS, overall survival; AUC, area under the curve; ROC, receiver operating characteristic.


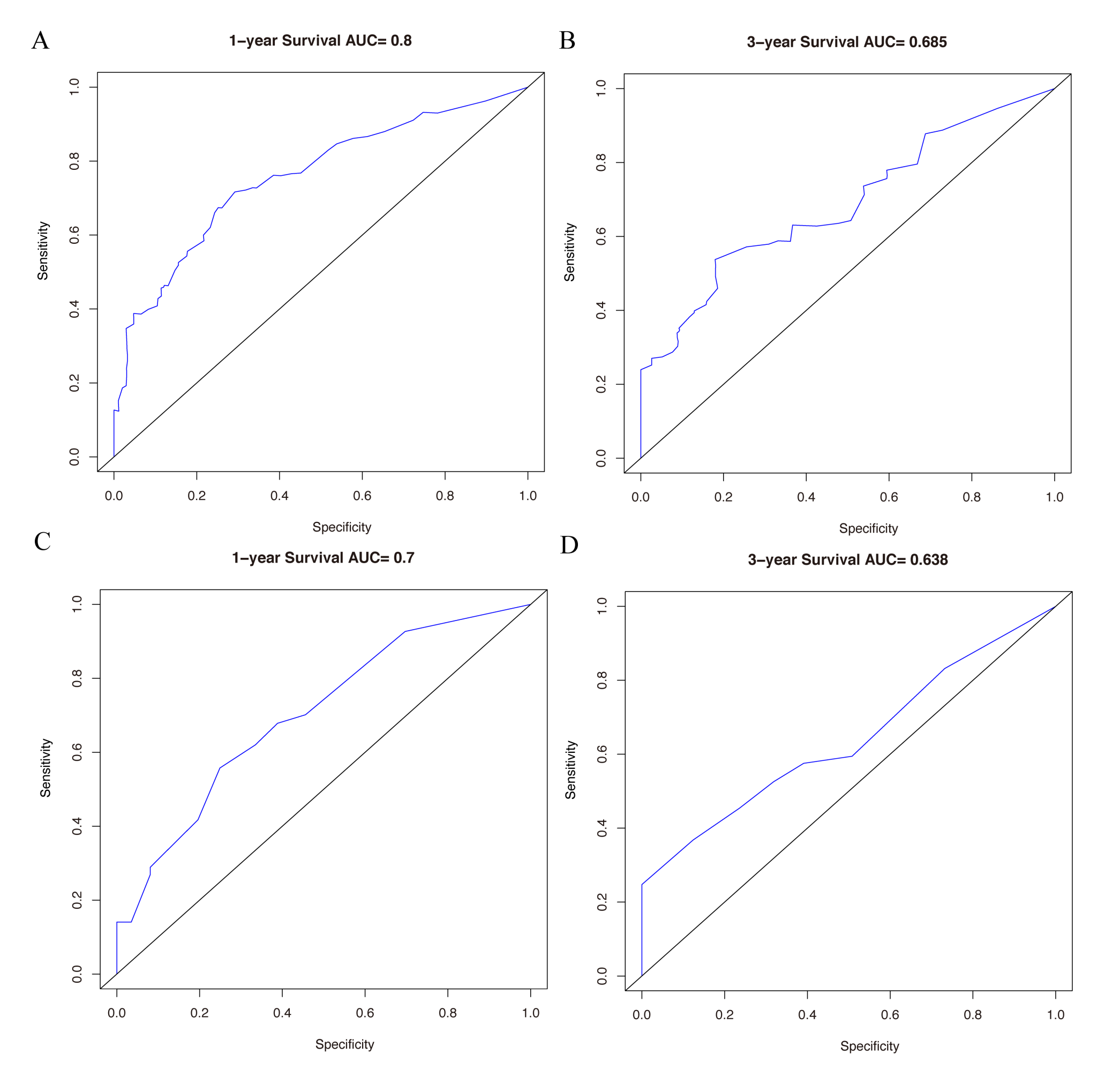


**Figure S3** The AUC values of ROC predicting for CSS: (A) 1-year survival rates of the nomogram in the training set; (B) 3-year survival rates of the nomogram in the training set; (C) 1-year survival rates of the nomogram in the validation set; (D) 3-year survival rates of the nomogram in the validation set. CSS, cancer-specific survival; AUC, area under the curve; ROC, receiver operating characteristic.


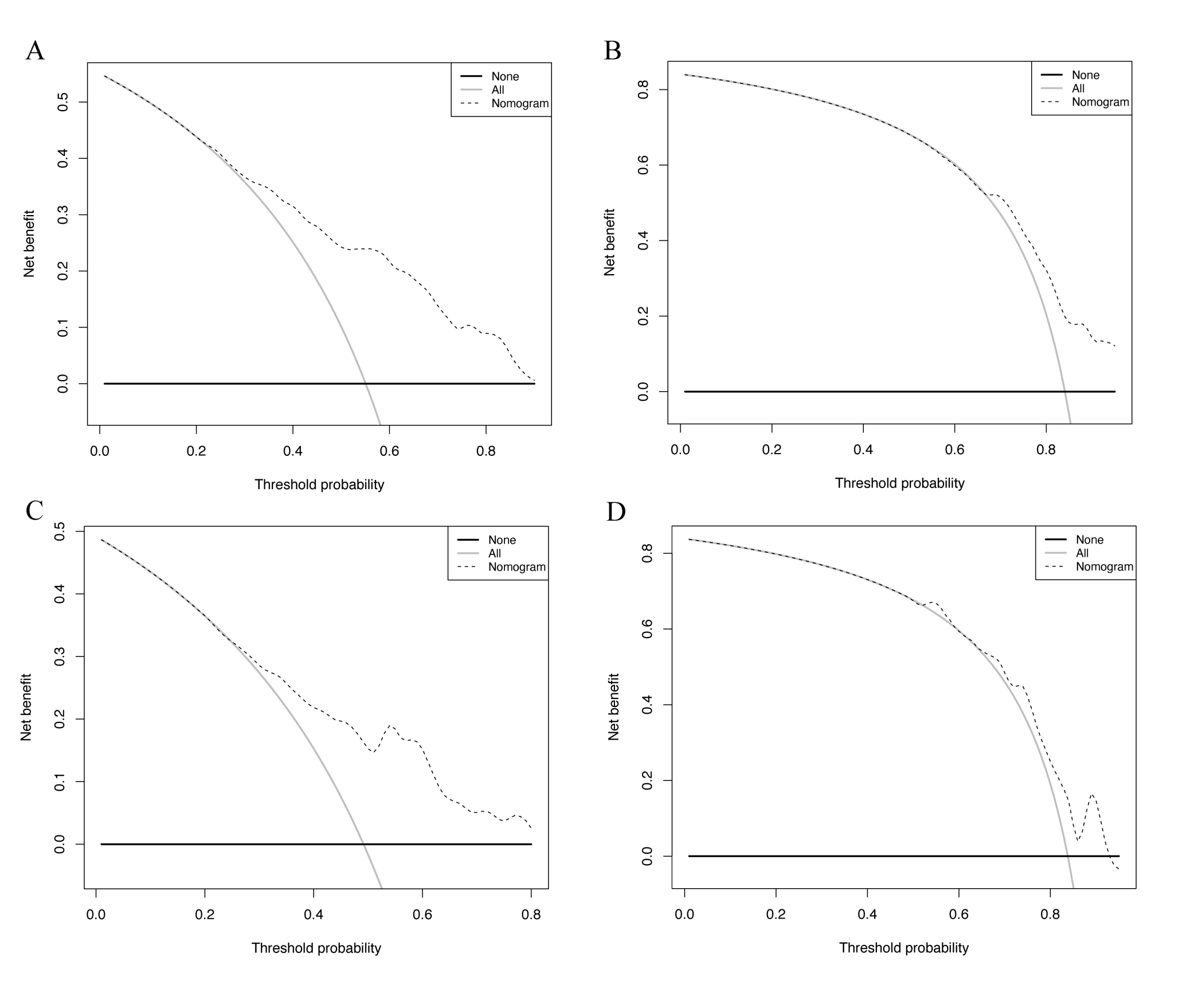


**Figure S4** Decision curve analysis for OS. A and B came from the training set; and C and D came from the validation set. The abscissa is the threshold probability, the ordinate is the net benefit rate. OS, overall survival.


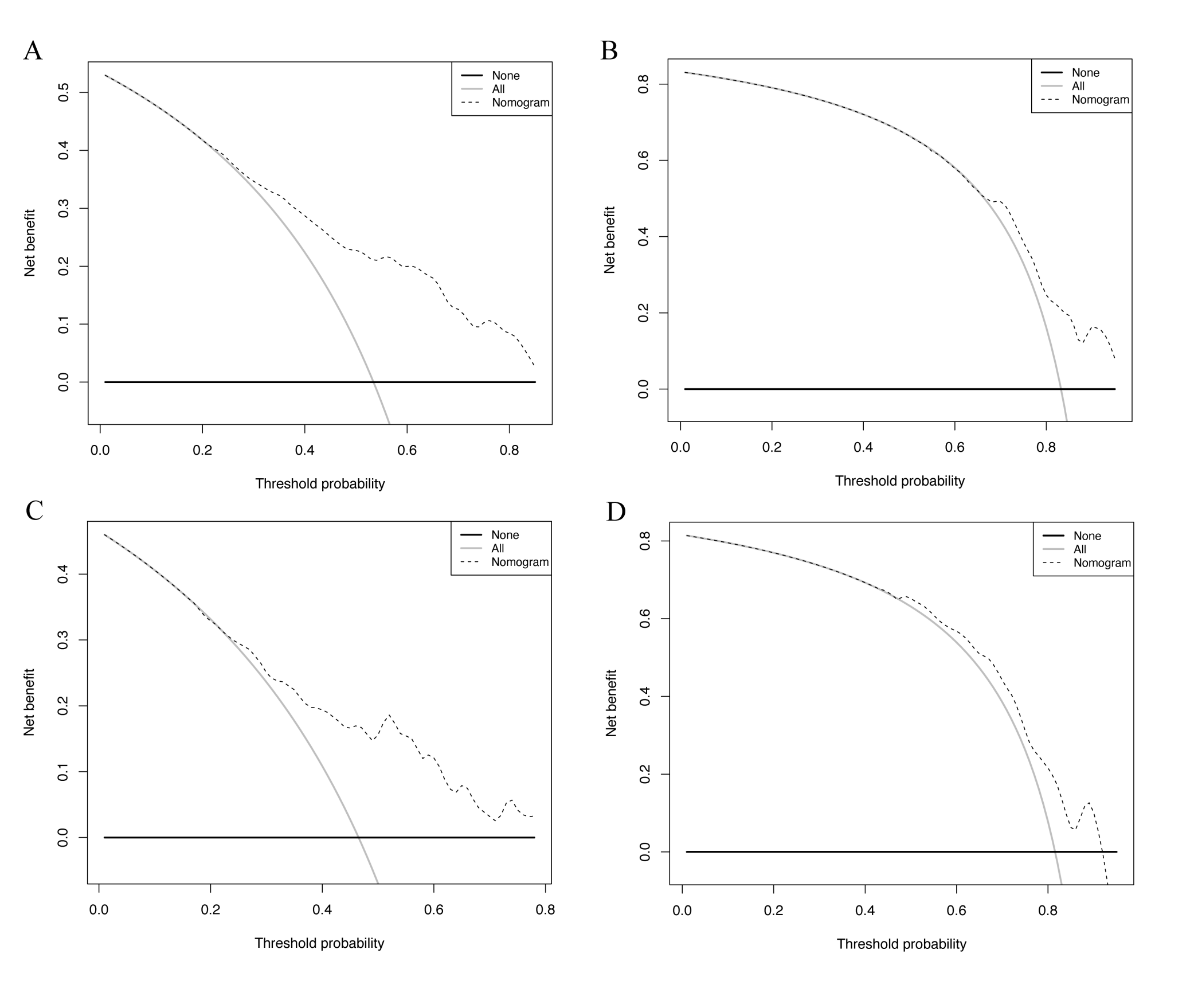


**Figure S5** Decision curve analysis for CSS. A and B came from the training set; and C and D came from the validation set. The abscissa is the threshold probability, the ordinate is the net benefit rate. CSS, cancer-specific survival.
